# Genome report: Genome of the Amazon guppy (*Poecilia bifurca*) reveals conservation of sex chromosomes and dosage compensation

**DOI:** 10.1093/g3journal/jkaf188

**Published:** 2025-08-19

**Authors:** Lydia J M Fong, Bernadette D Johnson, Iulia Darolti, Benjamin A Sandkam, Judith E Mank

**Affiliations:** Department of Zoology and Biodiversity Research Centre, The University of British Columbia, Vancouver, BC V6T 1Z4, Canada; Department of Zoology and Biodiversity Research Centre, The University of British Columbia, Vancouver, BC V6T 1Z4, Canada; Department of Ecology and Evolution, University of Lausanne, Vaud, Lausanne CH-1015, Switzerland; Department of Neurobiology and Behavior, Cornell University, Ithaca, NY 14853, United States; Department of Zoology and Biodiversity Research Centre, The University of British Columbia, Vancouver, BC V6T 1Z4, Canada

**Keywords:** *Poecilia bifurca*, amazon guppy, Y gene duplication, male germ cell expression, genome assembly

## Abstract

The Amazon guppy, *Poecilia bifurca*, is a small live-bearing fish. The close relatives *Poecilia reticulata, Poecilia picta*, and *Poecilia parae* all share the same sex chromosome system, but with substantial diversity in the degree of Y degeneration and the extent of X chromosome dosage compensation. In order to identify if *P. bifurca* shares the same sex chromosome system, we built a female (XX) draft genome with 55X coverage of PacBio HiFi data, resulting in a 785 Mb assembly with 94.4% BUSCO completeness. We used this genome and found that *P. bifurca* shares the same sex chromosomes as related species and shows substantial Y chromosome degeneration. We combined this with RNA-Seq data and found similar expression of X-linked genes between sexes, revealing that *P. bifurca* also exhibits complete X chromosome dosage compensation. We further identify 11 putative autosome-to-Y gene duplications, 5 of which show gene expression in guppy male germ cells.

## Introduction

Fishes in the genus *Poecilia* have been extensively studied in the context of sexual selection and sexual conflict (Endler 1980), life-history evolution (Meredith et al. 2011), and are an emerging model system for sex chromosome evolution. Several closely related species in the genus share the same sex chromosome system but with extensive differences in the extent of Y chromosome degeneration and X chromosome dosage compensation. *Poecilia reticulata* has homomorphic sex chromosomes with little degeneration of the Y chromosome in the region homologous to the X (Wright et al. 2017; Darolti et al. 2019; Du et al. 2025), while the nonrecombining region of the Y chromosome in its sister species, *Poecilia wingei*, has expanded substantially and is the largest chromosome in the genome (Nanda et al. 2014). *Poecilia parae* and *Poecilia picta* belong to a sister clade yet have heteromorphic sex chromosomes, extensive Y degeneration, and complete X chromosome compensation (Darolti et al. 2019; Metzger et al. 2021; Sandkam et al. 2021 ; Fong et al. 2023). Despite the difference in the state of Y chromosome degeneration and dosage compensation, these 4 species share a recent, single origin of their sex chromosome (Fong et al. 2023). This begs the question of why there is so much variation between species and what might we expect to observe in other members of this genus.

**Fig. 1.**
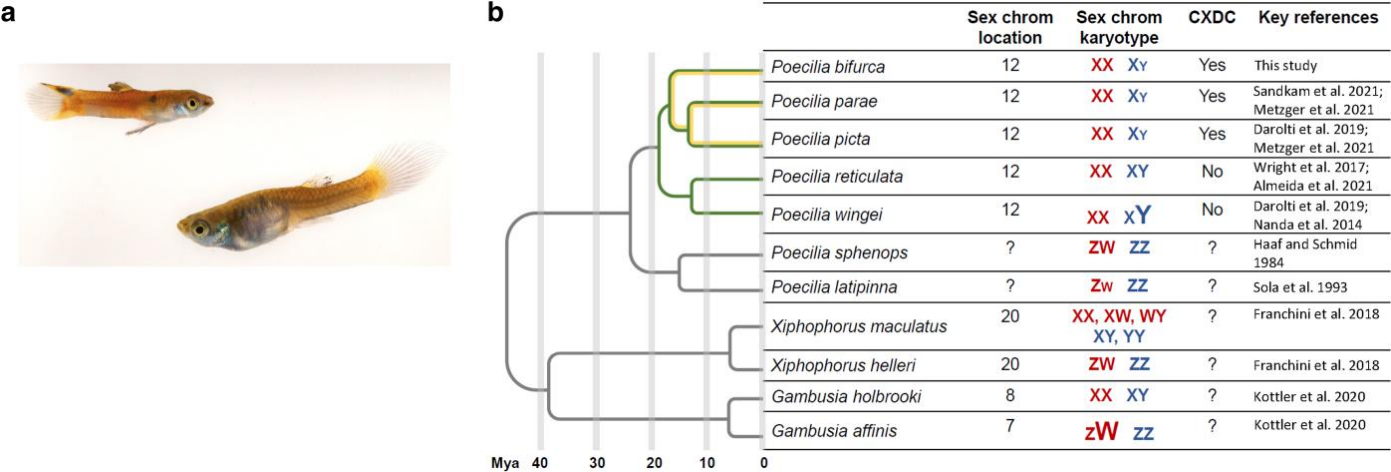
Photo of adult *P. bifurca* and the phylogeny of closely related species. a) A male individual is depicted on the left, a female on the right. b) Phylogeny from Rabosky et al. (2018) and Rabosky et al. (2013). Maximum likelihood origin of the sex chromosome system in *P. bifurca* and its close relatives is shown in green, and complete X chromosome dosage compensation (CXDC) shown in yellow. Included is the sex chromosome location on guppy chromosome number, relative size differences of the sex chromosomes with female karyotypes in red and male in blue, evidence of complete X chromosome dosage compensation, and references.

The Amazon guppy (*Poecilia bifurca*) is sexually dimorphic (Fig. 1a) and is in the same clade as *P. picta* and *P. parae* (Fig. 1b; Meredith et al. 2011; Rabosky et al. 2013, 2018; Pollux et al. 2014) but its ecology, including life history and mating behavior, are less studied compared to its close relatives (Rosen and Bailey 1963; Breden et al. 1999). Furthermore, it lacks a reference genome, and potential sex chromosomes have not been identified. We might predict that *P. bifurca* possesses a degenerated Y chromosome shared with *P. picta* and *P. parae*. However, frequent sex chromosome turnover (the old sex chromosomes revert to autosomes and a new set of sex chromosomes arise) has been observed among closely related fish species (Vicoso 2019), for example in sticklebacks (Ross et al. 2009), Lake Tanganyika cichlids (Gammerdinger and Kocher 2018; Roberts et al. 2009), and medaka (Myosho et al. 2015), suggesting that this is also a possibility for *P. bifurca*.

To address this question, we first built a draft female genome for *P. bifurca* using PacBio long-read sequencing of an individual female (XX). Using short read DNA data from males and females, we determined that *P. bifurca* shares the same sex chromosome with its close relatives (guppy chromosome 12), and that the Y chromosome is highly degenerated, similar to *P. picta* and *P. parae*. Using expression data from males and females, we show that *P. bifurca* also shares complete X chromosome dosage compensation with *P. picta* and *P. parae*. Finally, we use coverage estimates for both sexes and polymorphism data to identify 11 putative autosome-to-Y chromosome duplications and explore the expression of these loci.

## Materials and methods

### Sample collection and sequencing methods

*P. bifurca* individuals were obtained from Paramaribo, Suriname, and exported by PeZa Wildlife through the Suriname Department of Fisheries (certificate number 202011002E). All individuals were kept in temperature-controlled chambers at 26 to 28 °C, and tanks were kept at a pH range from 6.0 to 7.0. Individuals for sequencing were flash frozen in liquid nitrogen and stored in −80 °C until sample extraction. For genome assembly, high molecular weight (HMW) DNA was collected from 1 female (25.1 ng/μL) using the New England Biotechnological Lab Monarch Kit for PacBio Sequel II SMRT 8 M. HMW DNA fragments longer than 20 kb were used for CCS HiFi library prep, and sequencing was done using 1 SMRT cell to the resulting average genomic coverage was 55X.

For short-read DNA sequencing, we extracted DNA from 3 males and 3 females using the Qiagen DNeasy Blood and Tissue Kit (31.5 to 187.7 μg/μL). Libraries were prepared using a PCR-free method and sequenced on a single lane of a NovaSeq S4 PE150 flow cell, generating an average coverage of ∼62.3X (Supplementary Table 1). For RNA-sequencing, we collected somatic (tail and head) tissue from the same 3 adult males and 3 adult females, and used the Qiagen RNeasy Kit to extract total mRNA (RIN ≥ 8). Libraries were prepared using the NEB Ultra II Directional poly(A) mRNA kit and sequenced on a single lane of a NovaSeq SP flow cell for 150 bp paired-end reads (PE2 × 150 bp). Library preparations and sequencing were done at Toronto SickKids Hospital Sequencing Centre.

For both the DNA and RNA short read data, we followed the quality assessment protocol from Darolti et al. (2019). Briefly, sample quality for Illumina sequences was assessed with FastQC v.10.1 (Andrews 2010; http://www.bioinformatics.babraham.ac.uk/projects/fastqc/, last accessed on July 8, 2023). Adaptors were removed and trimmed using Trimmomatic v.0.36, using the universal adapter for RNA sequences with the commands LEADING: 3 TRAILING: 3 SLIDINGWINDOW:4:15 MINLEN:50, PHRED 33. DNA sequences were trimmed using TruSeq3-PE-2 adapter following the same commands.

### Genome assembly and repeat annotation

The PacBio sequences were corrected (i.e. consensus of overlapping reads), trimmed, and assembled using CANU v.2.2 (Koren et al. 2017) using a maximum error allotment of 4.5%, an estimated genome size of 750 Mb based on the female reference of *P. picta* (∼744 MB; Metzger et al. 2023), and all other parameters set to default. We then used Pilon v.1.22 (Walker et al. 2014) with *–fix all* and *–mindepth 0.5* to correct the long-read CANU assembly with the female *P. bifurca* Illumina DNA sequences. Finally, the draft genome assembly was oriented to the female *P. picta* reference genome (Metzger et al. 2023) using RagTag v.2.1.0 (Alonge et al. 2022) following the *ragtag_scaffold.py* script. We then ran BUSCO v.5.3.2 with the cyprinodontiformes_odb10 database (Simão et al. 2015) to check for genome assembly completeness (∼94.4%; Table 1). Lastly, we followed the Earl Grey (Baril et al. 2024) pipeline with default parameters to identify repeat content.

**Table 1.** Assembly statistics for the draft genome, its repeat content, and for the transcriptome.

	Parameter	Statistic
PacBio Assembly	Total Length (bp)	785,347,781
(Canu)	Contigs	2,492
	Largest contig (bp)	13,935,650
	N50 (bp)	3,301,456
	L50	63
BUSCO (%)	Completeness	94.4
	Missing	5.26
	Fragmented	0.31
	Complete and duplicated	9.29
	Complete and single copy	85.14
Repeat Content (%)	DNA Transposon	12.83
	LINE	7.88
	LTR Retrotransposon	5.22
	Other (Simple Repeat, Microsatellite, RNA)	3.60
	Penelope	0.19
	Rolling Circle	0.29
	SINE	0.09
	Unknown	8.02
	Total	38.12
Transcriptome	*de novo* Trinity Assembly (transcripts)	17,521
	*P. picta* BLAST hit (genes)	14,257

BUSCO results using the cyprinodontiformes_odb10 database are reported as percentages. Numbers reported in the repeat content represents percentage of the genome that is composed of the classified repeat DNA. The number of de novo transcripts assembled from Trinity and the number of transcripts that BLAST to the *P. picta* genome annotation are reported.

### Transcriptome assembly

We first used HISAT2 v.2.1.0 (Kim et al. 2019) to align the RNA sequences to our draft *P. bifurca* genome and then used Trinity v.2.11.0 (Grabherr et al. 2011) with default parameters to build our *de novo* transcriptome. Reads were combined from all samples, then we filtered the transcriptome by removing unpaired and discordant alignments using the built-in Trinity script *align_and_estimate_abundance.pl* that maps the *de novo* transcriptome using Bowtie2 (Langmead et al. 2009). We filtered the transcriptome noncoding RNA by removing transcripts with BLAST hits to the *Oryzias latipes* ncRNA database (Ensembl: ASM223467v1; Hunt et al. 2018). The transcriptome was further filtered to remove coding regions within transcripts without an open reading frame and those with open-reading frames smaller than 150 bp using TransDecoder v.5.5.0 (Haas et al. 2013; http://transdecoder.github.io, last accessed November 24, 2023) with default parameters. The TransDecoder contigs were further assembled with CAP3 (Huang and Madan 1999) to build the final transcriptome. Genes from the transcriptome were annotated using BLAST against the *P. picta* female genome annotation and using the top blast hit.

### Identifying the sex chromosomes and dosage compensation

Degeneration of the Y results in reduced read coverage of males compared to females for the X chromosome. To determine the location of the sex chromosomes and the extent of Y degeneration, we used the male and female Illumina DNA data to identify the sex chromosomes. First, we mapped short reads to the *P. bifurca* draft genome assembly using bwa *aln* and *samse* v0.6.1 (Li and Durbin 2009), then found unique mapping sites using grep “XT:A:U”. We then sorted the aligned reads using SAMTools v.1.3.1 (Li et al. 2009) and extracted the coverage for each individual using soap.coverage v.2.7.7 (https://bio.tools/soap; Li et al. 2008). We calculated the average coverage for males and females separately and then compared male to female (M:F) coverage (log_2_ average male coverage/log_2_ average female coverage). We also used Bowtie2 v.1.1.2 (Langmead et al. 2009) to map the reads of all samples to the draft genome assembly. We re-analyzed *P. picta* and *P. parae* using the same pipeline to compare sex chromosome coverage across the species. These samples included 3 female and 3 male *P. picta* individuals from Darolti et al. (2019) and 3 females and 3 males of the parae morph of *P. parae* from Sandkam et al. (2021). Finally, we used the HAWK v. 1.7.0 pipeline (Rahman et al. 2018) to count and identify male-specific 21 bp *k*-mers (*Y*-mers) for each species using counts from the Bonferroni-corrected file. We identified shared *Y*-mers with normalized coverage greater than 20X coverage (the threshold used to detect excess unique *Y*-mers relative to female-specific *k*-mers) across *P. bifurca*, *P. picta*, and *P. parae* to test for shared ancestry of the Y chromosome.

To determine the extent of X chromosome dosage compensation, we followed the approach of Darolti et al. (2019) to calculate allele-specific expression (ASE). First, we aligned the RNA-seq data to the *P. picta* female reference genome using STAR align v. 2.7.11 (Dobin et al. 2013) with default parameters and *–outFilterMultimapNmax 1*. We then called single-nucleotide polymorphisms (SNPs) for males and females separately using SAMTools v.1.3.1 (Li et al. 2009) *mpileup* and filtered the vcf file using VarScan v2.3.9 (Koboldt et al. 2009) *–min-coverage 2 –min-ave-qual 20 –min-freq-for-hom 0.90 –P-value 1 –strand-filter 0 –min-var-free 1e-10*. We removed clusters of more than 5 SNPs in 100 bp windows to avoid bias in our ASE estimations from the preferential assignment of reads to the reference allele and removed triallelic SNPs.

We expect most genes to have biallelic expression (equal expression of the alleles from both chromosomes) and thus expect ∼0.5 probability to recover reads from either chromosome. Therefore, we tested for ASE by identifying significant deviations from 0.5 using a 2-tailed binomial test (*P* < 0.05) on the final filtered SNP dataset. We called SNPs as ASE if a minimum of 70% of the reads came from 1 chromosome and kept identified genes with ASE that had at least 1 SNP with a consistent ASE pattern across all heterozygous samples. We then used a Chi-squared test examine differences in ASE patterns between the autosomes and the sex chromosomes in males and females. Finally, we used HTSeq v.2.0.5 (Putri et al. 2022) to count reads for the transcripts assembled from our de novo transcriptome and normalized the counts using gene length to get gene expression and compared M:F expression.

### Y gene duplication analysis

We followed the pipeline from Lin et al. (2022) to identify putative autosome-to-Y chromosome gene duplications using a combination of M:F F_ST_, M:F coverage, and M:F SNP density. Once duplicated from an autosome to the Y, the accumulation of Y-specific SNPs will produce elevated M:F F_ST_ and M:F SNP density > 1 (Bissegger et al. 2020; Tobler et al. 2017). Furthermore, complete autosome-to-Y gene duplication will produce M:F coverage = 1.5. Partial Y-gene duplicates will exhibit M:F coverage between 1 and 1.5 (Tobler et al. 2017; Lin et al. 2022; van der Bijl et al. 2025). To find loci with these characteristics, we first filtered the mapped reads for males and females from the above analysis and called SNPs using SAMTools/bcftools v.19 (Li 2011) *mpileup -Ou -q 20 -Q 20 –skip-indels -a FORMAT/AD,FORMAT/DP* then *call -mv -Oz -f GQ*. SNPs were then filtered using VCFtools v0.1.12b (Danecek et al. 2011) *–maf 0.05 –mac 1 –min-alleles 2 –max-alleles 2 –max-missing 0.9 –min-meanDP 10 –max-meanDP 100 –minGQ 25* and specifying coding regions identified from the Trinity assembled transcriptome.

We used the *–weir-fst-pop* function from VCFtools v0.1.12b (Danecek et al. 2011) to identify the M:F F_ST_ for each SNP in the coding regions. Next, we used the *–site-depth* function to identify the gene read depth for males and females. We used the resulting read depth files to run the *gene_coverage.py* script from Lin et al. (2022) to calculate the M:F coverage of the genes and acquire the corresponding M:F F_ST_. Finally, we calculated M:F SNP density for the putative Y-gene duplications using the sam2pro pipeline (Haubold et al. 2010; http://guanine.evolbio.mpg.de/mlRho/, last accessed September 09, 2024). The M:F read depth of the genes followed a normal distribution, therefore we used 2 standard deviations to identify genes that have an increased M:F read depth for partial duplicates (>1.206). We bootstrapped the M:F F_ST_ of autosomal genes to calculate the 95% confidence intervals (CI). We called genes that had M:F coverage >1.206 and M:F F_ST_ values greater than the 95% CI (>0.196) as putative Y-gene duplications.

### Gene expression of the putative Y gene duplications

Because expression in the male germ cells is often associated with autosome-to-Y gene duplications (Kaessmann 2010), we assessed expression of putative duplicated genes in *P. reticulata*, the nearest species for which single-cell expression data are available. Gene-by-cell count matrices from a previously collected single-cell RNA sequencing (scRNA seq) dataset on *P. reticulata* gonads were used to provide expression profiles for male gonad-specific cells (Darolti and Mank 2023). Briefly, gonads were dissected from reproductively mature male guppies and used to generate 3 nonoverlapping pools with 5 individuals each. An estimated 8,000 cells from each sample were used to prepare 3′ single-cell libraries using the 10X Genomics Chromium Next GEM Single Cell 3′ kit v3.1. Libraries were sequenced on an Illumina NovaSeq 6000 sequencer, with an average sequencing depth of 20,000 read pairs per cell. CellRanger v5.0.1 (Zheng et al. 2017) was used to build a reference index using the *P. reticulata* genome (Ensembl: GCA_000633615.2; Hunt et al. 2018), align reads and identify cell-associated barcodes to generate gene-by-cell count matrices. Artefactual doublet cells were detected and removed using DoubletFinder v2.0.3 (McGinnis et al. 2019).

Cells were filtered and their counts used for downstream analyses, based on the following quality control thresholds: number of unique molecular identifiers (UMI) per cell (nUMI ≥ 500), number of detected genes per cell (nGene ≥ 250), the log-transformed ratio of genes per UMI (log10GenesPerUMI > 0.80), and the proportion of transcripts mapping to mitochondrial genes (mitoRatio < 0.20). Following quality filtering, cells were clustered into distinct cell types, which were labeled based on known marker gene information (Jiang et al. 2021; Wang et al. 2023). Genes with low expression—defined as having fewer than 1 count in at least 10 cells—were removed. Gene counts were summed across cells within each cluster to generate a pseudo-bulk expression matrix.

This pseudo-bulk matrix was input into the R package edgeR v4.4.2 (R Development Core Team 2024) to create a DGEList object. Normalization factors were calculated using the trimmed mean of *M*-values (TMM) method to scale raw library sizes *calcNormFactors(DGEList, method =* “*TMM*”*)* (Robinson and Oshlack 2010). The resulting dataset contained TMM-normalized counts per million values for genes expressed in spermatids and spermatocytes cell types of the *P. reticulata* male gonad. We used these normalized count values to determine cell-specific expression of our putatively Y duplicated genes.

## Results and discussion

### Genome assembly and transcriptome

Our PacBio sequencing resulted in 41,971,990,442 bp in total, with mean read length of 12.68 Kb, and mean barcode quality of 82 (Supplementary Table 1). Our assembled de novo genome was ∼785 Mb (Table 1), comparable to the genomes of *P. reticulata* (731.6 Mb; Künstner et al. 2016) and *P. picta* (744 Mb; Metzger et al. 2023). The CANU assembly of the genome had 2,492 contigs with contig coverage ∼37X, an NG50 of 3.3 Mb, with contig size ranging up to ∼14 Mb (Table 1). 50% of the assembly resides on 63 contigs (LG50; Table 1).

After orienting the assembly to the *P. picta* female reference genome, we recovered 36 scaffolds with successful alignment to all 23 *P. picta* chromosomes. Our draft genome had a BUSCO completeness of 94.4% and contained ∼38% repetitive DNA, as compared to the 29.55% repetitive DNA reported in the *P. picta* reference genome (Metzger et al. 2023; Table 1). The BUSCO score is slightly lower than that reported for *P. picta* (97.65%; Metzger et al. 2023), likely due to the different databases used (cyprinodontiformes_odb10 database in this analysis, eukaryota_odb10 database in Metzger et al. 2023).

We recovered an average of 79.7 million RNA reads per sample (Supplementary Table 1). Following the de novo transcriptome assembly, we found a total of 17,521 transcripts and 14,257 of these transcripts mapped to the *P. picta* reference genome (Table 1). Moving forward, we focused only on the transcripts that had the top BLAST hit to *P. picta* genes.

### Identifying the sex chromosomes and dosage compensation

**Fig. 2.**
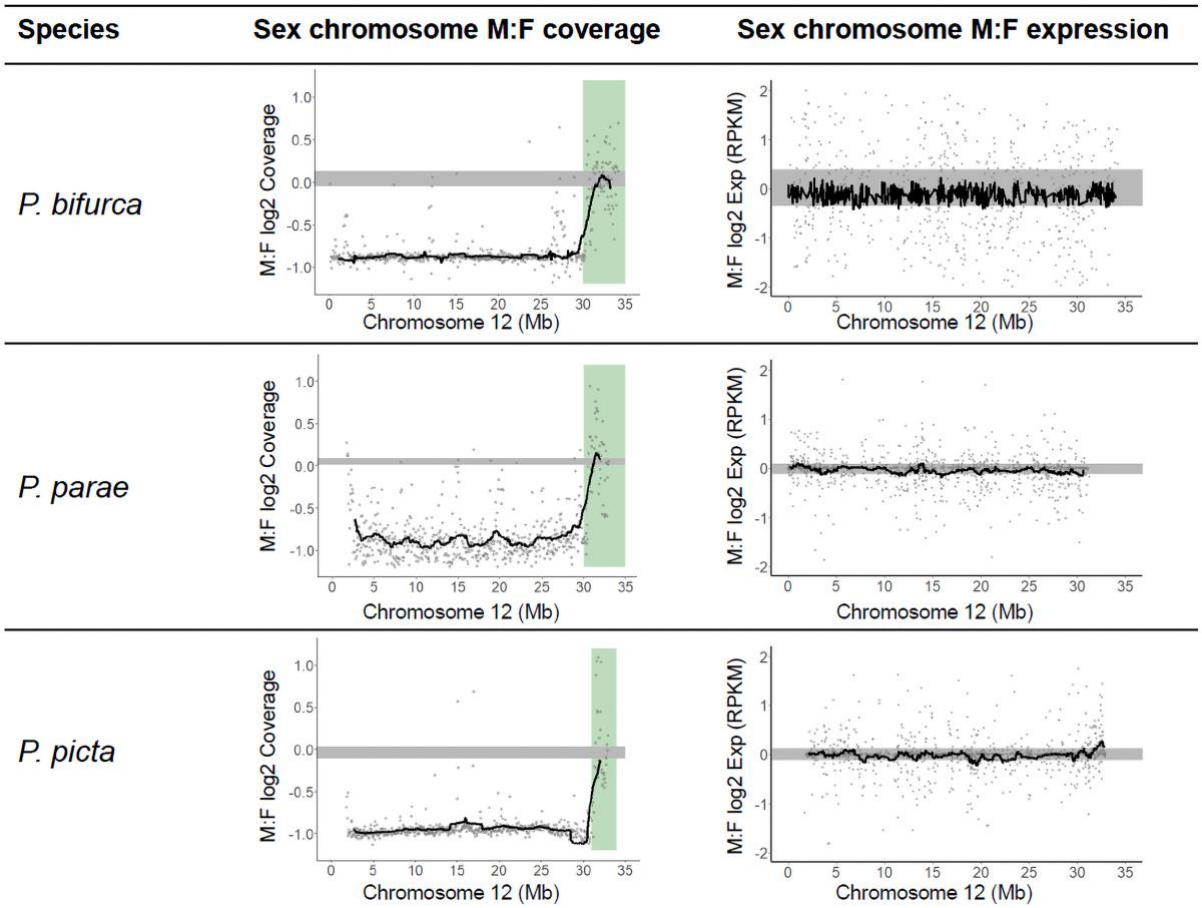
Characterizing the sex chromosome in *P. bifurca* and its closest relatives. Male:female (M:F) log_2_ fold change of reads mapped to chromosome 12, with the value of 0.0 representing equal coverage. The green (vertical) bar in the middle panel represents the pseudoautosomal region (PAR). The right panel shows the M:F log_2_ fold change of the gene expression of chromosome 12. *P. picta* and *P. parae* reads were mapped to the *P. picta* female reference genome, and *P. bifurca* reads were mapped to the draft *P. bifurca* genome. The black line represents the sliding window with the window size of 50 kb. The gray bar represents the 95% CI calculated from 1,000 bootstraps of the M:F log_2_ fold change of the autosomes in the respective panels.

We recovered an average of 156.0 million Illumina reads per sample, resulting in an average 62.3X coverage (Supplementary Table 1). Using a female (XX) genome, we expect Y degeneration to result in reduced M:F coverage (Darolti et al. 2019), and this approach revealed the *P. bifurca* sex chromosomes corresponds to guppy chromosome 12. Chromosome 12 had a log_2_ M:F coverage = −1.0, spanning 0 to 30 Mb, suggesting widespread Y degeneration of this region (Fig. 2). There is a small region, 30–34Mb, with equal coverage between males and females (log_2_ M:F ≈0.0), likely representing the pseudoautosomal region where recombination still occurs between the X- and Y-chromosome (Fig. 2). The significant decrease in male coverage is not observed elsewhere in the genome (Supplementary Fig. 1). When compared to the log_2_ M:F coverage of *P. picta* and *P. parae* chromosome 12, we see similar patterns of Y degeneration along the same region of the chromosome (Fig. 2).

**Fig. 3.**
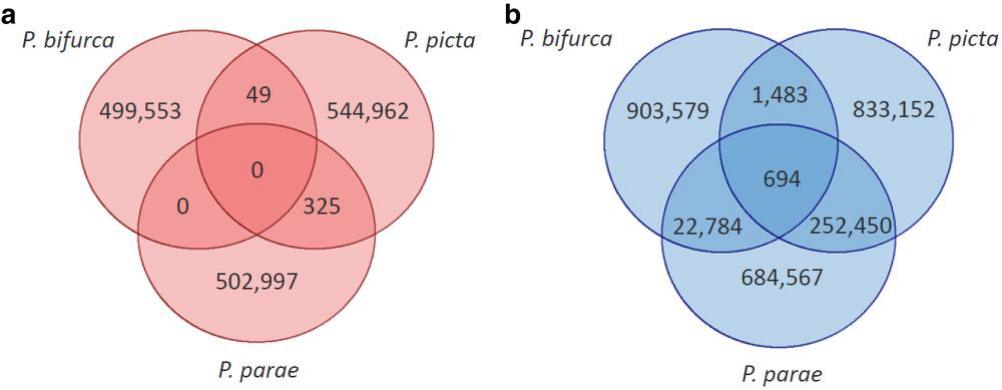
Shared sex-specific *k*-mers between *P. bifurca*, *P. picta*, and *P. parae*. a) Female-specific *k*-mers found unique in the species and shared between species, representing false positives. b) Male-specific *k*-mers (*Y*-mers) found unique in the species and shared between species. There are at least 30X more shared *Y*-mers than false positives.

We further tested for shared ancestry of the Y chromosome among *P. picta, P. parae*, and *P. bifurca* by comparing shared male-specific sequences known as Y-mers (Carvalho and Clark 2013; Torres et al. 2018; Kabir et al. 2022). We found more Y-mers are shared between the 3 species than expected by chance (measured by the female-specific k-mers), suggesting shared ancestry of the sex chromosome (Fig. 3).

Although degeneration of Y chromosome gene content leaves males with 1 functional copy of X-linked genes compared to the 2 copies present in females, males in *P. picta* and *P. parae* exhibit similar expression of the X in males and females, consistent with complete X chromosome dosage compensation (Darolti et al. 2019; Metzger et al. 2021, 2023). To test for complete dosage compensation in *P. bifurca*, we compared M:F gene expression on chromosome 12 and found no difference in gene expression between males and females, similar to patterns observed in *P. picta* and *P. parae* and indicative of complete dosage compensation (Fig. 2).

**Fig. 4.**
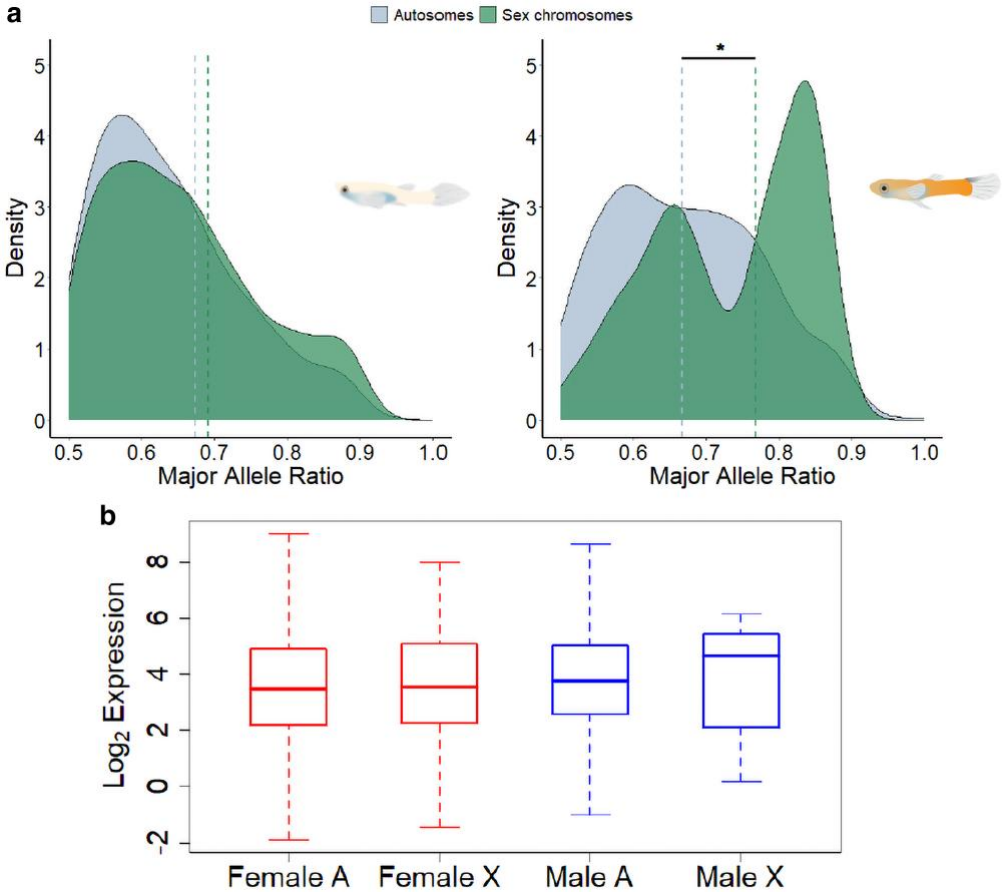
Allele-specific expression (ASE) of *P. bifurca*. a) The density distribution of female (left) and male (right) major allele ratio (frequency) in autosomes (blue-grey) and the sex chromosomes (green). The dashed lines represent the median major allele ratio of each respective distribution. The median value of autosome and sex chromosome major allele ratio in males is statistically significant (*χ*² test; *P* = 0.0276). b) Boxplots show the average female (red) and average male (blue) normalized expression between autosomes (A) and the non-recombining region of the sex chromosome genes with an ASE pattern (X) in *P. bifurca*, with no statistically significant difference between the autosomes and X in either sex (*χ*² test; female *P* = 0.493; male *P* = 0.482).

Complete X chromosome dosage compensation is further corroborated by the ASE distribution, identified by the frequency distribution of the major allele ratio. Female *P. bifurca* show a similar ASE pattern between autosomes and sex chromosomes with no difference in the median major allele ratio (*P* = 1; Fig. 4a), whereas males have a preferential expression from 1 allele in their sex chromosome (median = 0.767) compared to the autosomes (median = 0.667; *P* = 0.0276; Fig. 4a). Moreover, gene expression between the autosomes and non-recombining region of the sex chromosomes in females (*P* = 0.493) and males (*P* = 0.482) do not differ from each other (Fig. 4b).

### Putative Y gene duplications

Following Lin et al. (2022), we identified putative autosome-to-Y gene duplications, finding 11 autosomal genes with elevated M:F read depth, elevated M:F F_ST_, and M:F SNP density ≥1, distributed across 8 of the 22 autosomes (Table 2). We used GeneCards (Stelzer et al. 2016) and Gene Ontology Annotation (Ashburner et al. 2000; Carbon et al. 2021) to identify the function of these genes, where known.

**Table 2.** Putative autosome to Y-gene duplications and their autosomal location in *P. bifurca*.

Gene Name	Chrom	Mean M:F FST	M:F Read Depth	M:F SNP Density	Gene Function	Expr. in spermatid (CPM)	Expr. in spermatocyte (CPM)
*slc4a10*	LG2	0.3333	1.4561	1.0000	Transporter activity; solute:inorganic anion antiporter activity	N/A	N/A
*mapk6*	LG3	0.3333	1.2185	1.0288	Transferase activity; transferring phosphorus-containing groups; protein tyrosine kinase activity	62.7700	48.3394
gcshb*	LG3	0.5000	1.2079	1.1312	Enzyme binding; aminomethyltransferase activity	N/A	N/A
*trub2*	LG5	0.5000	1.2059	1.6863	Pseudouridine synthase activity	29.1664	15.4314
*ddi2*	LG5	0.3182	1.2362	1.0726	Aspartic-type endopeptidase activity; regulation of DNA stability; and regulation of protein stability.	N/A	N/A
*spsb3*	LG8	0.3182	1.2727	1.0532	SPRY domain-containing SOCS box protein 3	N/A	N/A
*ccdc97*	LG8	0.3333	1.4407	1.1852	Coiled-coil domain	71.5283	73.7606
*cfap100*	LG10	0.5000	1.2326	1.4808	Cilia and flagella associated protein 100	21.8748	21.0428
*sdc3*	LG11	0.5385	1.2086	1.0000	Cytoskeletal protein binding	N/A	N/A
*ablim3*	LG14	0.3333	1.2688	1.0000	Actin binding	8.2903	2.1043
*slc7a8*	LG18	0.5000	1.3852	1.5556	Peptide antigen binding; antiporter activity	N/A	N/A

Average male:female (M:F) read depth, M:F F_ST_, and M:F SNP density for the coding regions of the gene are reported. Gene functions are referenced from gene ontology (Ashburner et al. 2000; Carbon et al. 2021) and GeneCards (Stelzer et al. 2016). Expression reported is the median of all male samples in CPM if applicable. The gene marked with an * is the top BLAST hit from *P. reticulata* to an uncharacterized gene from the *P. picta* genome annotation.

We tested the assumption that genes that are expressed in male germ cells are more likely to be duplicated on the Y chromosome and passed on in the male germline (Connallon and Clark 2010; Kaessmann 2010). To do so, we looked at the gene expression of the putative Y-genes in *P. reticulata* spermatocyte and spermatid cells (male germ cell types). We found 5 of the 11 genes had expression in the male gonadal tissues (Table 2). Interestingly, all of the putative Y-gene duplicated genes are expressed in at least 1 human male testes cell type (Supplementary Table 2).

## Conclusion

In this study, we reveal the conservation of the sex chromosomes in *P. bifurca* and complete X chromosome dosage compensation. Through the use of different sequencing technologies, we built a draft genome with 94.4% completeness and found 81.4% transcript matches to its close relative, *P. picta*. Furthermore, we identified 11 putative autosome-to-Y gene duplications. Our genome is a resource for future research, especially work that uses a comparative approach to better understand the ecology and evolution of *Poecilia* fishes.

## Supplementary Material

jkaf188_Supplementary_Data

## Data Availability

All *P. bifurca* sequence data have been deposited under NCBI BioProject PRJNA1259976. The draft *P. bifurca* genome is available under NCBI BioSample SAMN48384351. Data for *P. picta* (Darolti et al. 2019) and *P. parae* (Sandkam et al. 2021) used for re-analysis of coverage and expression can be found under NCBI BioProject ID PRJNA528814 and BioProject PRJNA714257, respectively. The genome annotation for *P. bifurca* is available at GSA FigShare: https://doi.org/10.25387/g3.29120048. All scripts and related codes can be found at https://github.com/ljmfong/Poecilia-bifurca-Characterizing-Sex-Chromosome. Supplemental material available at G3 online.

## References

[jkaf188-B1] Almeida P et al 2021. Divergence and remarkable diversity of the Y chromosome in guppies. Mol Biol Evol. 38:619–633. doi: 10.1093/molbev/msaa257. 33022040 PMC7826173

[jkaf188-B2] Alonge M et al 2022. Automated assembly scaffolding using RagTag elevates a new tomato system for high-throughput genome editing. Genome Biol. 23:258. doi: 10.1186/s13059-022-02823-7. 36522651 PMC9753292

[jkaf188-B3] Andrews S . 2010. *FastQC: a quality control tool for high throughput sequence data*. https://www.bioinformatics.babraham.ac.uk/projects/fastqc/.

[jkaf188-B4] Ashburner M et al 2000. Gene ontology: tool for the unification of biology. Nat Genet. 25:25–29. doi: 10.1038/75556. 10802651 PMC3037419

[jkaf188-B5] Baril T, Galbraith J, Hayward A. 2024. Earl Grey: a fully automated user-friendly transposable element annotation and analysis pipeline. Mol Biol Evol. 41:msae068. doi: 10.1093/molbev/msae068. 38577785 PMC11003543

[jkaf188-B6] Bissegger M, Laurentino TG, Roesti M, Berner D. 2020. Widespread intersex differentiation across the stickleback genome–The signature of sexually antagonistic selection?. Mol Ecol. 29:262–271. doi: 10.1111/mec.15255. 31574563

[jkaf188-B7] Breden F, Ptacek MB, Rashed M, Taphorn D, Figueiredo CA. 1999. Molecular phylogeny of the live-bearing fish genus *Poecilia* (Cyprinodontiformes: *Poeciliidae*). Mol Phylogenet Evol. 12:95–104. doi: 10.1006/mpev.1998.0600. 10381313

[jkaf188-B8] Carbon S et al 2021. The gene ontology resource: enriching a GOld mine. Nucleic Acids Res. 49:D325–D334. doi: 10.1093/nar/gkaa1113. 33290552 PMC7779012

[jkaf188-B9] Carvalho AB, Clark AG. 2013. Efficient identification of Y chromosome sequences in the human and *Drosophila* genomes. Genome Res. 23:1894–1907. doi: 10.1101/gr.156034.113. 23921660 PMC3814889

[jkaf188-B10] Connallon T, Clark AG. 2010. Gene duplication, gene conversion and the evolution of the Y chromosome. Genetics. 186:277–286. doi: 10.1534/genetics.110.116756. 20551442 PMC2940292

[jkaf188-B11] Danecek P, et al 2011. The variant call format and VCFtools. Bioinform. 27:2156–2158. doi: 10.1093/bioinformatics/btr330. PMC3137218 21653522

[jkaf188-B12] Darolti I et al 2019. Extreme heterogeneity in sex chromosome differentiation and dosage compensation in livebearers. Proc Natl Acad Sci U S A. 116:19031–19036. doi: 10.1073/pnas.1905298116. 31484763 PMC6754558

[jkaf188-B13] Darolti I, Mank JE. 2023. Sex-biased gene expression at single-cell resolution: cause and consequence of sexual dimorphism. Evol Lett. 7:148–156. doi: 10.1093/evlett/qrad013. 37251587 PMC10210449

[jkaf188-B14] Dobin A et al 2013. STAR: ultrafast universal RNA-seq aligner. Bioinformatics. 29:15–21. doi: 10.1093/bioinformatics/bts635. 23104886 PMC3530905

[jkaf188-B15] Du K et al 2025. Identification of the male-specific region on the guppy Y chromosome from a haplotype-resolved assembly. Genome Res. 35:489–498. doi: 10.1101/gr.279582.124. 40044220 PMC11960691

[jkaf188-B16] Endler JA . 1980. Natural selection on color patterns in *Poecilia reticulata*. Evolution. 34:76–91. doi: 10.2307/2408316. 28563214

[jkaf188-B17] Fong LJM et al 2023. Evolutionary history of the *Poecilia picta* sex chromosomes. Genome Biol Evol. 15:evad030. doi: 10.1093/gbe/evad030. 36802329 PMC10003743

[jkaf188-B18] Franchini P et al 2018. Long-term experimental hybridisation results in the evolution of a new sex chromosome in swordtail fish. Nat Commun. 9:5136. doi: 10.1038/s41467-018-07648-2. 30510159 PMC6277394

[jkaf188-B19] Gammerdinger WJ, Kocher TD. 2018. Unusual diversity of sex chromosomes in African cichlid fishes. Genes (Basel). 9:480. doi: 10.3390/genes9100480. 30287777 PMC6210639

[jkaf188-B20] Grabherr MG, et al 2011. Trinity: reconstructing a full-length transcriptome without a genome from RNA-Seq data. Nat Biotechnol. 29:644–652. doi: 10.1038/nbt.1883. 21572440 PMC3571712

[jkaf188-B21] Haaf T, Schmid M. 1984. An early stage of ZW/ZZ sex chromosome differentiation in *Poecilia sphenops* var. *melanistica* (*Poeciliidae*, cyprinodontiformes). Chromosoma. 89:37–41. doi: 10.1007/BF00302348.

[jkaf188-B22] Haas BJ et al 2013. *De novo* transcript sequence reconstruction from RNA-Seq using the Trinity platform for reference generation and analysis. Nat Protoc. 8:1494–1512. doi: 10.1038/nprot.2013.084. 23845962 PMC3875132

[jkaf188-B23] Haubold B, Pfaffelhuber P, Lynch M. 2010. mlRho–a program for estimating the population mutation and recombination rates from shotgun-sequenced diploid genomes. Mol Ecol. 19:277–284. doi: 10.1111/j.1365-294X.2009.04482.x. 20331786 PMC4870015

[jkaf188-B24] Huang X, Madan A. 1999. CAP3: a DNA sequence assembly program. Genome Res. 9:868–877. doi: 10.1101/gr.9.9.868. 10508846 PMC310812

[jkaf188-B25] Hunt SE et al 2018. Ensembl variation resources. Database. 2018:bay119. doi: 10.1093/database/bay119. 30576484 PMC6310513

[jkaf188-B26] Jiang M et al 2021. Characterization of the zebrafish cell landscape at single-cell resolution. Front Cell Dev Biol. 9:2734. doi: 10.3389/fcell.2021.743421. PMC8517238 34660600

[jkaf188-B27] Kabir A et al 2022. Repeated translocation of a supergene underlying rapid sex chromosome turnover in *Takifugu* pufferfish. Proc Natl Acad Sci U S A. 119:e2121469119. doi: 10.1073/pnas.2121469119. 35658077 PMC9191631

[jkaf188-B28] Kaessmann H . 2010. Origins, evolution, and phenotypic impact of new genes. Genome Res. 20:1313–1326. doi: 10.1101/gr.101386.109. 20651121 PMC2945180

[jkaf188-B30] Kim D, Paggi JM, Park C, Bennett C, Salzberg SL. 2019. Graph-based genome alignment and genotyping with HISAT2 and HISAT-genotype. Nat Biotechnol. 37:907–915. doi: 10.1038/s41587-019-0201-4. 31375807 PMC7605509

[jkaf188-B31] Koboldt DC et al 2009. VarScan: variant detection in massively parallel sequencing of individual and pooled samples. Bioinformatics. 25:2283–2285. doi: 10.1093/bioinformatics/btp373. 19542151 PMC2734323

[jkaf188-B32] Koren S et al 2017. Canu: scalable and accurate long-read assembly via adaptive *k* -mer weighting and repeat separation. Genome Res. 27:722–736. doi: 10.1101/gr.215087.116. 28298431 PMC5411767

[jkaf188-B33] Kottler VA et al 2020. Independent origin of XY and ZW sex determination mechanisms in mosquitofish sister Species. Genetics. 214:193–209. doi: 10.1534/genetics.119.302698. 31704715 PMC6944411

[jkaf188-B34] Künstner A et al 2016. The genome of the Trinidadian guppy, *Poecilia reticulata*, and variation in the Guanapo population. PLoS One. 11:e0169087. doi: 10.1371/journal.pone.0169087. 28033408 PMC5199103

[jkaf188-B35] Langmead B, Trapnell C, Pop M, Salzberg SL. 2009. Ultrafast and memory-efficient alignment of short DNA sequences to the human genome. Genome Biol. 10:R25. doi: 10.1186/gb-2009-10-3-r25. 19261174 PMC2690996

[jkaf188-B36] Li H et al 2009. The sequence alignment/map format and SAMtools. Bioinformatics. 25:2078–2079. doi: 10.1093/bioinformatics/btp352. 19505943 PMC2723002

[jkaf188-B37] Li H . 2011. A statistical framework for SNP calling, mutation discovery, association mapping and population genetical parameter estimation from sequencing data. Bioinformatics. 27:2987–2993. doi: 10.1093/bioinformatics/btr509. 21903627 PMC3198575

[jkaf188-B38] Li H, Durbin R. 2009. Fast and accurate short read alignment with burrows–wheeler transform. Bioinformatics. 25:1754–1760. doi: 10.1093/bioinformatics/btp324. 19451168 PMC2705234

[jkaf188-B39] Li R, Li Y, Kristiansen K, Wang J. 2008. SOAP: short oligonucleotide alignment program. Bioinformatics. 24:713–714. doi: 10.1093/bioinformatics/btn025. 18227114

[jkaf188-B40] Lin Y et al 2022. Gene duplication to the Y chromosome in Trinidadian guppies. Mol Ecol. 31:1853–1863. doi: 10.1111/mec.16355. 35060220

[jkaf188-B41] McGinnis CS, Murrow LM, Gartner ZJ. 2019. DoubletFinder: doublet detection in single-cell RNA sequencing data using artificial nearest neighbors. Cell Syst. 8:329–337.e4. doi: 10.1016/j.cels.2019.03.003. 30954475 PMC6853612

[jkaf188-B42] Meredith RW, Pires MN, Reznick DN, Springer MS. 2011. Molecular phylogenetic relationships and the coevolution of placentotrophy and superfetation in *Poecilia* (*Poeciliidae*: Cyprinodontiformes). Mol Phylogenet Evol. 59:148–157. doi: 10.1016/j.ympev.2011.01.014. 21292015

[jkaf188-B43] Metzger DCH et al 2023. Transposon wave remodeled the epigenomic landscape in the rapid evolution of X-chromosome dosage compensation. Genome Res. 33:1917–1931. doi: 10.1101/gr.278127.123. 37989601 PMC10760456

[jkaf188-B44] Metzger DCH, Sandkam BA, Darolti I, Mank JE. 2021. Rapid evolution of complete dosage compensation in *Poecilia*. Genome Biol Evol. 13:evab155. doi: 10.1093/gbe/evab155. 34240180 PMC8325565

[jkaf188-B45] Myosho T, Takehana Y, Hamaguchi S, Sakaizumi M. 2015. Turnover of sex chromosomes in *Celebensis* group medaka fishes. G3 (Bethesda). 5:2685–2691. doi: 10.1534/g3.115.021543. 26497145 PMC4683641

[jkaf188-B46] Nanda I et al 2014. Sex chromosome polymorphism in guppies. Chromosoma. 123:373–383. doi: 10.1007/s00412-014-0455-z. 24676866

[jkaf188-B47] Pollux BJA, Meredith RW, Springer MS, Garland T, Reznick DN. 2014. The evolution of the placenta drives a shift in sexual selection in livebearing fish. Nature. 513:233–236. doi: 10.1038/nature13451. 25043015

[jkaf188-B48] Putri GH, Anders S, Pyl PT, Pimanda JE, Zanini F. 2022. Analysing high-throughput sequencing data in python with HTSeq 2.0. Bioinformatics. 38:2943–2945. doi: 10.1093/bioinformatics/btac166. 35561197 PMC9113351

[jkaf188-B49] Rabosky DL et al 2013. Rates of speciation and morphological evolution are correlated across the largest vertebrate radiation. Nat Commun. 4:1958. doi: 10.1038/ncomms2958. 23739623

[jkaf188-B50] Rabosky DL et al 2018. An inverse latitudinal gradient in speciation rate for marine fishes. Nature. 559:392–395. doi: 10.1038/s41586-018-0273-1. 29973726

[jkaf188-B51] Rahman A, Hallgrímsdóttir I, Eisen M, Pachter L. 2018. Association mapping from sequencing reads using k-mers. Elife. 7:e32920. doi: 10.7554/eLife.32920. 29897334 PMC6044908

[jkaf188-B52] R Development Core Team . 2024. R: a language and environment for statistical computing. R Foundation for Statistical Computing.

[jkaf188-B53] Roberts RB, Ser JR, Kocher TD. 2009. Sexual conflict resolved by invasion of a novel sex determiner in Lake Malawi cichlid fishes. Science. 326:998–1001. doi: 10.1126/science.1174705. 19797625 PMC3174268

[jkaf188-B54] Robinson MD, Oshlack A. 2010. A scaling normalization method for differential expression analysis of RNA-seq data. Genome Biol. 11:R25. doi: 10.1186/gb-2010-11-3-r25. 20196867 PMC2864565

[jkaf188-B55] Rosen DE, Bailey RM. 1963. The Poeciliid fishes (Cyprinodontiformes), their structure, zoogeography, and systematics (vol. 126). Bulletin of the American Museum of Natural History.

[jkaf188-B56] Ross JA, Urton JR, Boland J, Shapiro MD, Peichel CL. 2009. Turnover of sex chromosomes in the stickleback fishes (*Gasterosteidae*). PLoS Genet. 5:e1000391. doi: 10.1371/journal.pgen.1000391. 19229325 PMC2638011

[jkaf188-B57] Sandkam BA et al 2021. Extreme Y chromosome polymorphism corresponds to five male reproductive morphs of a freshwater fish. Nat Ecol Evol. 5:939–948. doi: 10.1038/s41559-021-01452-w. 33958755

[jkaf188-B58] Simão FA, Waterhouse RM, Ioannidis P, Kriventseva EV, Zdobnov EM. 2015. BUSCO: assessing genome assembly and annotation completeness with single-copy orthologs. Bioinformatics. 31:3210–3212. doi: 10.1093/bioinformatics/btv351. 26059717

[jkaf188-B59] Sola L, Bressanello S, Rasch EM, Monaco PJ. 1993. Cytogenetics of bisexual/unisexual species of *Poecilia*. IV. Sex chromosomes, sex chromatin composition and ag-NOR polymorphisms in *Poecilia latipinna*: a population from Mexico. Heredity (Edinb). 70:67–71. doi: 10.1038/hdy.1993.9.

[jkaf188-B60] Stelzer G, et al 2016. The GeneCards suite: from gene data mining to disease genome sequence analyses. Curr Protoc Bioinformatics. 54:1–30. 10.1002/cpbi.5. 27322403

[jkaf188-B61] Tobler R, Nolte V, Schlötterer C. 2017. High rate of translocation-based gene birth on the *Drosophila* Y chromosome. Proc Natl Acad Sci U S A. 114:11721–11726. doi: 10.1073/pnas.1706502114. 29078298 PMC5676891

[jkaf188-B62] Torres MF et al 2018. Genus-wide sequencing supports a two-locus model for sex-determination in *Phoenix*. Nat Commun. 9:3969. doi: 10.1038/s41467-018-06375-y. 30266991 PMC6162277

[jkaf188-B63] van der Bijl W et al 2025. Deep learning reveals the complex genetic architecture of male guppy colouration. Nat Ecol Evol. 1–12. 10.1101/2023.09.29.560175. 40596731

[jkaf188-B64] Vicoso B . 2019. Molecular and evolutionary dynamics of animal sex-chromosome turnover. Nat Ecol Evol. 3:1632–1641. doi: 10.1038/s41559-019-1050-8. 31768022

[jkaf188-B65] Walker BJ, et al 2014. Pilon: an integrated tool for comprehensive microbial variant detection and genome assembly improvement. PLoS ONE. 9:e112963. doi: 10.1371/journal.pone.0112963. 25409509 PMC4237348

[jkaf188-B66] Wang R et al 2023. Construction of a cross-species cell landscape at single-cell level. Nucleic Acids Res. 51:501–506. doi: 10.1093/nar/gkac633. 35929025 PMC9881150

[jkaf188-B67] Wright AE et al 2017. Convergent recombination suppression suggests role of sexual selection in guppy sex chromosome formation. Nat Commun. 8:14251. doi: 10.1038/ncomms14251. 28139647 PMC5290318

[jkaf188-B68] Zheng GXY et al 2017. Massively parallel digital transcriptional profiling of single cells. Nat Commun. 8:14049. doi: 10.1038/ncomms14049. 28091601 PMC5241818

