# Supplementary material for: Genome report: Genome of the Amazon guppy (Poecilia bifurca) reveals conservation of sex chromosomes and dosage compensation
Source: G3 (Bethesda). 2025 Aug 19;15(10):jkaf188. doi: 10.1093/g3journal/jkaf188 (PMC12506654; doi:10.1093/g3journal/jkaf188)
Supplement: jkaf188_Supplementary_Data [file jkaf188_supplementary_data.docx]

**Supplemental Table 1. Summary statistics of sequenced *P. bifurca* individuals.**

| Individual | Trimmed RNA (Mb) | Trimmed DNA (Mb) | DNA Coverage | PacBio HMW DNA |
| --- | --- | --- | --- | --- |
| Female 1 | 86.8 | 159.7 | 64X |  |
| Female 2 | 77.6 | 148.1 | 59X | Read length: 12,679  Number of bases: 41,971,990,442  Barcode quality: 82 |
| Female 3 | 76.0 | 145.2 | 58X |  |
| Male 1 | 70.0 | 152.9 | 61X |  |
| Male 2 | 83.0 | 150.2 | 60X |  |
| Male 3 | 85.6 | 179.7 | 72X |  |

**Supplemental Table 2.**  **Expression of putative autosome-to-Y duplicated genes in human testes cells.**

|  | Expression (nTPM)^1^ | | | |
| --- | --- | --- | --- | --- |
| Gene Name | Spermatogonia | Spermatocytes | Early Spermatids | Late Spermatids |
| *slc4a10* | n.s. | n.s | 22.4 | 10.4 |
| *mapk6* | 76.0 | 62.6 | 94.8 | 225.7 |
| *gcshb** | 67.0 | 17.3 | 11.7 | 8.5 |
| *trub2* | 20.3 | 14.6 | 12.9 | 16.0 |
| *ddi2* | 8.6 | 10.6 | 1.7 | n.s |
| *spsb3* | 41.3 | 21.7 | 31.1 | 92.7 |
| *ccdc97* | 12.9 | 20.0 | 4.1 | 6.5 |
| *cfap100* | 1.3 | 4.7 | 226.4 | 750.4 |
| *sdc3* | n.s | n.s | 7.3 | 81.3 |
| *ablim3* | 2.3 | 2.3 | 1.6 | 1.2 |
| *slc7a8* | 1.0 | n.s | 2.1 | 2.5 |

*gcsh in humans.

^1^ From Human Protein Atlas: Karlsson et al. 2021. **A single-cell type transcriptomics map of human tissues.** *Sci Adv.* 7(31): eabh2169.


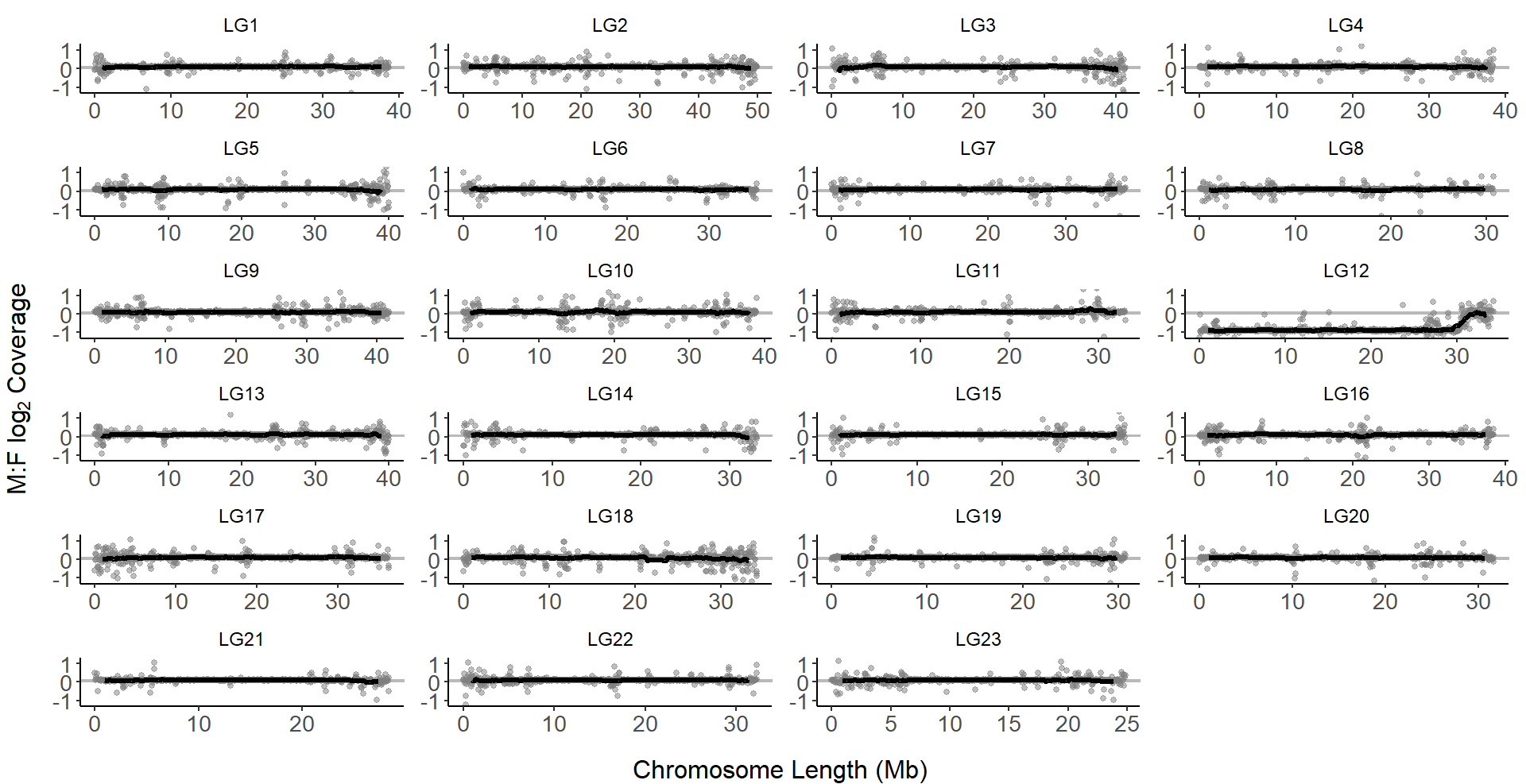


**Supplemental Figure 1. The M:F log_2_ fold change in coverage for all chromosomes in *P. bifurca*.** Male and female reads were mapped to the *P. bifurca* draft genome, with the value of 0.0 representing equal coverage. Each panel represents the respective chromosome (LG). The black line represents the sliding window with the window size of 50kb. The grey bar represents the 95% CI calculated from 1000 bootstraps of the M:F log_2_ fold change of the autosomes.

**References**

Karlsson, M., Zhang, C., Loren, M., Zheng, W., Digre, A., Katona, B., Sjöstedt, E., Butler, L., Odeberg, J., Dusart, P., Edfors, F., Oksvold, P., von Feilitzen, K., Zwahlen, M., Arif, M., Altay, O., Li, X., Ozcan, M., Mardinoglu, A., … Lindskog, C. 2021. A single–cell type transcriptomics map of human tissues. *Science Advances, 7*(31):eabh2169. doi: https://doi.org/10.1126/sciadv.abh2169.
